# Upcycling of face masks to application-rich multi- and single-walled carbon nanotubes

**DOI:** 10.1007/s42823-022-00398-8

**Published:** 2022-09-30

**Authors:** Varun Shenoy Gangoli, Thomas Mahy, Tim Yick, Yubiao Niu, Richard E. Palmer, Alvin Orbaek White

**Affiliations:** 1 Energy Safety Research Institute, Swansea University Bay Campus, Swansea, SA1 8EN UK; 2 Department of Chemical Engineering, Swansea University Bay Campus, Swansea, SA1 8EN UK; 3 Nanomaterials Lab, Mechanical Engineering, Faculty of Science and Engineering, Swansea University, Bay Campus, Swansea, SA1 8EN UK

**Keywords:** Carbon, Carbon nanotube, Electrical conductor, Face masks, Green chemistry, Recycling

## Abstract

We report the use of face mask materials as a carbon precursor for the synthesis of multi- and single-walled carbon nanotubes (CNTs) in an open-loop chemical recycling process. Novel surgical mask precursors were suspended in toluene and injected into a chemical vapor deposition reactor previously optimized for CNT production using liquid injection. The CNTs were collected and characterized using resonant Raman spectroscopy, transmission electron microscopy (TEM) and scanning electron microscopy (SEM) before being turned into fibrils that were tested for electrical conductance. Once confirmed and repeated for statistical accuracy, a CNT-based Ethernet cable was manufactured and tested using iPerf3 for uplink and downlink speeds exceeding broadband standards worldwide. Radial breathing modes from Raman spectroscopy indicate single walled CNTs (SWCNTs) with diameters ranging from 0.8 to 1.55 nm and this matches well with TEM observations of SWCNTs with 1.5 nm diameter. This work pushes the horizon of feedstocks useful for CNT and SWCNT production in particular; this work demonstrates upcycling of materials fated for disposal into materials with positive net value and plenty of real-world applications.

**Supplementary Information:**

The online version contains supplementary material available at 10.1007/s42823-022-00398-8.

## Introduction

Managing the growing carbon waste from face masks is critical to stemming the tide of planetary desecration. Face masks, be they disposable or reusable, have been the subject of many discussions worldwide from the onset of the COVID-19 pandemic. In April 2020, China reported an average daily production rate of 450 million masks [1], and Taiwan is estimated to have produced and used 1.3 billion face masks in 3 months ending the same time [2] which translates to nearly 5500 metric tons of spent face masks being discarded after a single use. The WHO estimated a need for 45 million disposable face masks monthly in May 2020 [3], and all these estimates still paled to the estimated 52 billion disposable face masks that were produced in the entirety of 2020 [4], of which 1.5–2 billion have been conservatively calculated to have entered the oceans. A peer-reviewed study published in June 2020 [5] extrapolated a monthly consumption of 129 billion face masks in addition to 65 billion gloves, and all these numbers have added up for the remainder of 2020 through May 2022. Therefore, managing all the newly generated plastic waste from spent face masks is critical, especially as single-use masks have far outweighed re-usable/home-made masks in terms of efficacy and distribution [6].

Tackling the growing issue of waste management from personal protective equipment (PPE) has been ongoing [7–9], with various reports describing the utilization of face masks in the construction industry [10], sound absorption [11], and general engineering products [12]. Researchers at the Energy Safety Research Institute in Swansea University have previously reported the use of spent face masks for improved ferrous metallurgy [13]. Another work from the same research group described manufacturing novel face masks that aim to be re-usable next-gen surgical masks [14]. Scope for improvement and recycling remains, yet there is hope in the form of open-loop chemical recycling to convert facemasks (and other carbon waste) into functional materials and devices.

Carbon nanotubes (CNTs) have been a growing research topic and an industrial material for the better part of 30 years now, with many applications already realized in industries ranging from energy, transportation, manufacturing, construction, and even art [15–19]. We have previously reported the production of CNTs from paper [20] and waste plastics [21], which led to expertise in the dispersion of various plastics in organic solvents [22] that then have been demonstrated to produce electrically conductive CNTs used in the manufacturing of Ethernet cables [23] and audio cables [24]. The natural extension of this work was to use day-to-day accessible objects such as face masks as the plastic source for CNT synthesis and this work describes the results of our findings accordingly by pushing the boundaries of what could be used as a carbon source for CNT production, including single-walled carbon nanotubes.

## Experimental section

### Materials

**Fig. 1:**
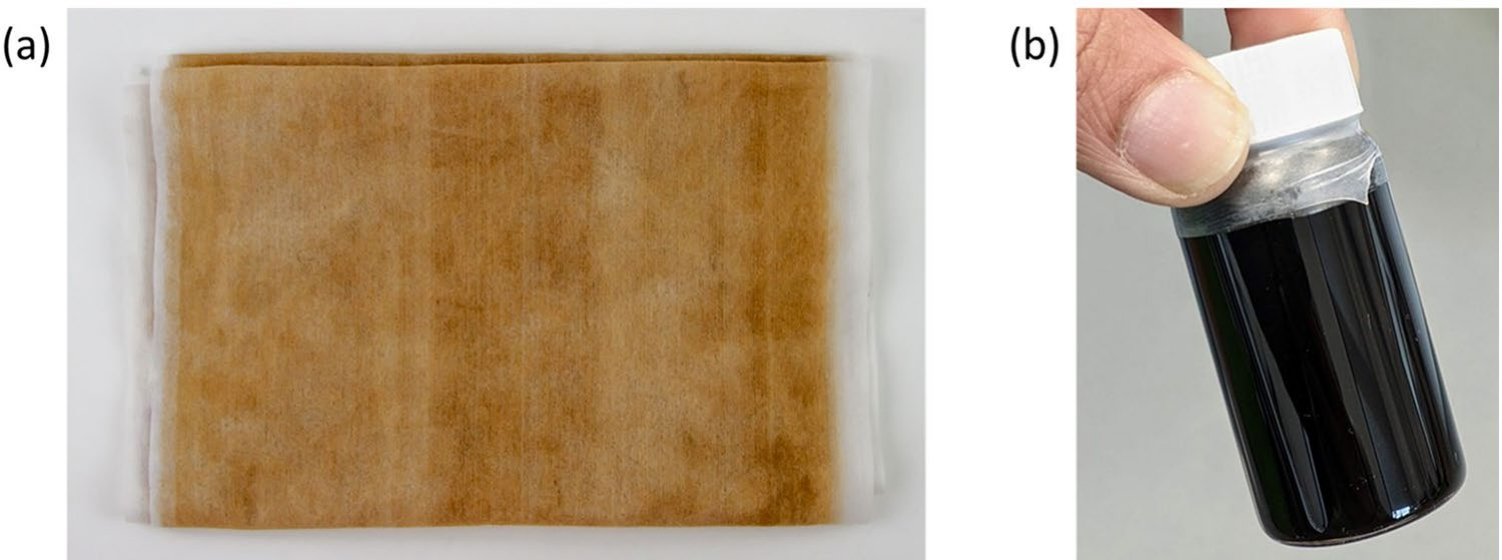
**a** Photograph of a section of face mask material used for CNT production. Sections were cut and **b** dispersed in toluene to form the plastic carbon precursor seen in a vial

The face mask materials were produced by the Barron Research Group as described before [14, 25]. This material was coarsely cut into smaller pieces (roughly 1 cm^2^ pieces) and added to anhydrous toluene [98% (C_6_H_5_CH3) Sigma Aldrich (U.K.)] for a final concentration of 10 wt% (w/w) inside a 100 mL round bottomed flask that was heated to 85 °C in an inert gas environment [99.998% pure nitrogen from B.O.C., Surrey, UK] with constant stirring overnight. The bulk majority of the dissolution happened within the first 30 min; however, it was prudent to ensure a longer contact time for maximum dispersion. The materials were found to be fully dispersed and then collected into 20 mL vials for storage and further use (Fig. 1).

The carbon nanotubes used in this work were produced using floating catalyst chemical vapor deposition (FC-CVD) in a two-zone horizontal furnace liquid injection reactor (LIR), with details described previously [21]. Despite the face mask material already containing iron catalyst in the form of ferroxane on the polyester, control experiments with simply the mask dispersion in toluene showed little CNT growth. Accordingly, ferrocene [98% (C_10_H_10_Fe) Sigma Aldrich (U.K.)] was added to the dispersion to have a net catalyst concentration of 5 wt% (w/w). One mL of this dispersion was injected into the reactor using a 16 AWG needle at a flow rate of 10 mL/h under a gas flow of 1 L/min using a blended carrier gas of composition 5 vol% hydrogen in nitrogen [BOC, Surrey, U.K.] into a two-zone horizontal furnace [Nanotech Innovations, Oberlin, OH, USA]. The first zone, used for catalyst formation and cracking of the carbon source, was set to approximately 450 °C and zone two, employed for CNT growth, was set to approximately 780 °C [26]. The CNTs were grown in a 78 cm long quartz tube with an outer diameter of 38 mm and inner diameter 36 mm [Multi-Lab, Newcastle Upon Tyne, U.K.]. All materials were used as received without prior cracking or drying and handled as described here [27].

### CNT characterization

The as-grown CNTs were imaged using scanning electron microscopy (SEM) to examine the product morphology, as well as to gauge the nature of the nanotubes to be single- vs. multi-walled. To do this, some of the CNTs were suspended in ethanol and drop cast onto a piece of clean silicon wafer for easier imaging. The CNT concentration was accounted for when imaging the product to allow examination of the entire ensemble as well as individual CNTs. The SEM (JEOL 7800F FEG from JEOL, Akishima, Tokyo, Japan) was used at 5 kV operating voltage or lower and with a working distance of approximately 10 mm.

Transmission electron microscopy (TEM) was also performed to support the SEM findings and better identify the presence of single- and multi-walled carbon nanotubes. It was conducted with a Thermo Fisher Scientific Talos F200X Transmission Electron Microscope with high-resolution TEM mode operating at 200 kV. The TEM samples were prepared by dipping holey carbon TEM grids into the as-produced CNT powders.

Subsequently, resonant Raman spectroscopy was conducted using a Renishaw inVia™ Raman microscope (Renishaw plc, Miskin, Pontyclun, UK) equipped with 633 nm and 785 nm lasers and a Leica PL Fluortar L50x/0.55 long working distance objective lens. The spectra were collected for the CNTs placed on a clean silicon wafer from 100 to 3200 cm^−1^ Raman shift. The laser beam was focused by maximizing the G-peak at ~ 1600 cm^−1^ intensity to get the optimal z-height alignment of the beam between the sample and the detector. For each CNT sample, a Raman spectrum was acquired in three separate locations for statistical accuracy.

Some of the CNTs, as buckypaper [23], were packed tightly into a 3 cm long piece of heat shrink tubing to make for a larger scale sample that should plausibly have continuous electron transmission pathways. To confirm this, the sample was tested using 2-point probe measurements conducted on an MPI TS50 manual probe station (MPI Corporation, Hsinchu, Taiwan) fitted with an MP40 micropositioner and 4 µm diameter tungsten tips. The probe station was connected to a Tektronix 2651A single channel source meter unit (Tektronix UK Ltd., Berkshire, U.K.) for both voltage supply and current readout, which in turn allowed the calculation of electrical resistance at the supplied voltage.

### Preparation and testing the CNT Ethernet cable

**Fig. 2:**
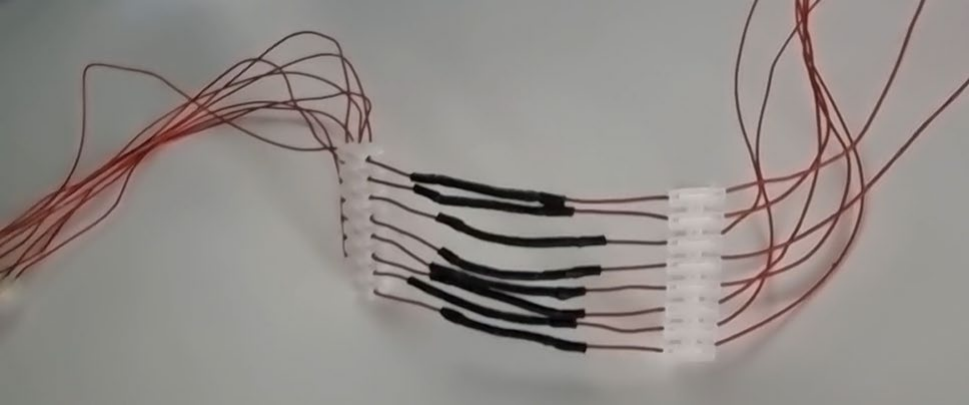
A close-up photograph of the CNT Ethernet cable to show the CNTs in the middle packed tightly inside heat shrink tubing and copper wiring on either side to form a power and data pathway from one end to another with CNTs in between. The ends of the wiring were crimped into RJ45 connectors to form the Ethernet cable

CNT product, as powder, was used to manufacture individual wires as described before [23, 24]. In short, the CNTs were firmly packed into the sheath of heat shrink tubing and copper wire was inserted into either end followed by compression for increased CNT-Cu contact. A heat gun was then used to shrink the outer sheath to size, as seen in Fig. 2, and the copper leads were crimped into pins to then insert into retail purchased RJ45 connectors (RS Components, Corby, UK) for testing as an Ethernet cable. The cable was then tested for performance efficacy using iPerf3 (ESnet/Lawrence Berkeley National Laboratory, USA) by directly connecting two computers, both running the same Windows 10 OS build, going from a Realtek 8125B 2.5 G LAN adapter (Realtek Semiconductor Corp., Hsinchu, Taiwan) capable for two-way traffic (server) to an Intel Killer E3100X LAN adapter (Intel, Santa Clara, CA, USA) as the client. Both the server and the client were individually capable of transfer speeds of up to 2500 Mbps, meaning that the CNT Ethernet cable was not being held back by other items in the chain. A standard 10 s/10 run test was performed to first authenticate the data transfer and next to measure the transfer speeds from the server (uplink) to the client (downlink) through the cable. The test was repeated for statistical accuracy, performed again using just a copper cable manufactured the same way but without the CNTs in the middle as a control, and the results tabulated for discussion and comparison.

## Results and discussion

### CNT characterization

**Fig. 3:**
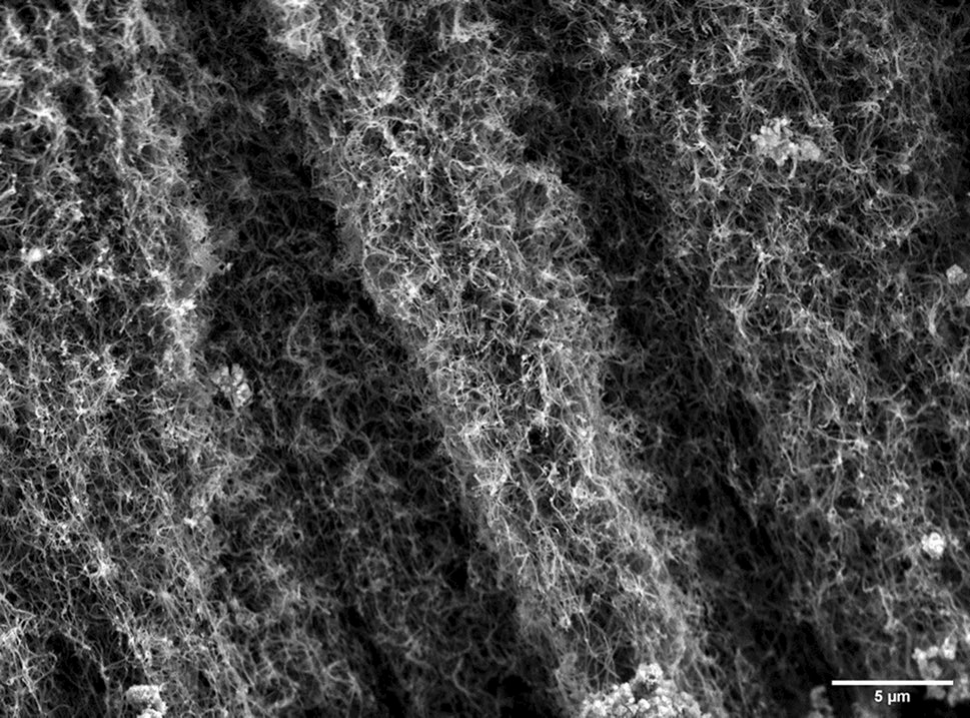
Scanning electron microscopy of CNTs produced using face mask materials dissolved in toluene as the carbon source and ferrocene as a source of iron catalyst. Residual catalyst and amorphous carbon impurities are present here, although most of the product remains CNTs

Scanning electron microscopy imaging, as seen in Fig. 3, confirms the product is predominantly carbon nanotubes. There is also some residual iron catalyst and amorphous carbon present, although CNTs comprise the bulk majority of the product. These are indeed multi-walled carbon nanotubes with some residual catalyst and amorphous carbon present [21], with typical morphology seen in Fig. 3. We have previously reported [23] findings that show the CNTs are produced predominantly from the dissolved plastics rather than toluene/ferrocene, and a control experiment was performed using simply toluene and ferrocene without any face masks dissolved while keeping everything else the same. The product yield was found to be lower at 0.32 g for the control experiment vs. 0.56 g with the face masks. Mass balance is similar to our previous reports [23] and showed a maximum CNT yield of ~ 10.5% for the control case as compared to 18.4% when grown using the face masks. SEM imaging done of the product from the control experiment (ESI figure S1) also reveals predominantly amorphous carbon as opposed to CNTs here, further showing the effective growth of CNTs directly from the face masks.

**Fig. 4:**
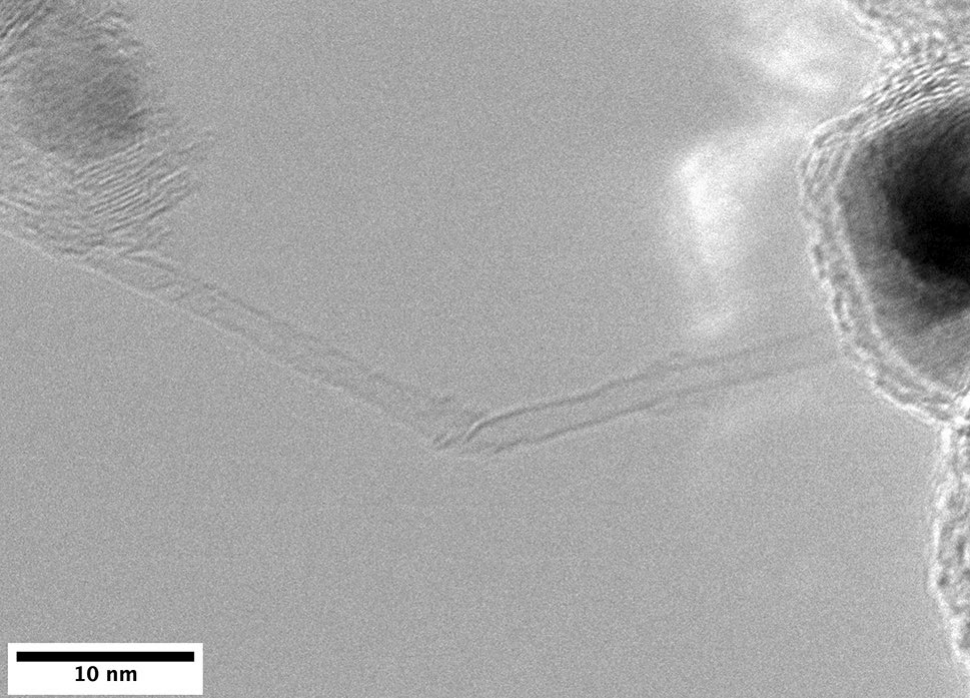
Transmission electron microscopy of CNTs produced using face mask materials dissolved in toluene as the carbon source and ferrocene as a source of iron catalyst. A mixture of single- and multi-walled CNTs are observed here in addition to residual catalyst and amorphous carbon

**Fig. 5:**
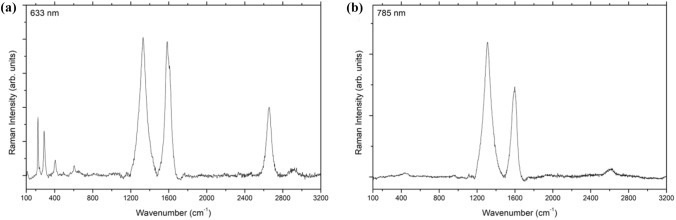
Resonant Raman spectroscopy of CNTs recorded using **a** 633 nm and **b** 785 nm lasers

Transmission electron microscopy corroborates these findings by showing a mixture of single- and multi-walled nanotubes, as seen in Fig. 4. The diameter of the SWCNT seen here was estimated to be 1.5 nm using ImageJ, with the MWCNTs ranging from 15 to 45 nm. Residual catalyst and some amorphous carbon is also observed here, again matching the results from SEM imaging.

resonant Raman spectroscopy confirmed the presence of CNTs again, and the presence of some radial breathing modes detected at 633 nm (Fig. 5a) further supported the presence of some single-walled CNTs [28–30] based on control experiments done to corroborate the absence of iron oxide peaks that could also be present here. The diameter of the CNTs corresponding to these RBM peaks can be calculated [28, 29] to be between 0.8 and 1.55 nm, which is in line with the diameter of the SWCNT seen in Fig. 4. The peak at ~ 1350 cm^−1^ confirms a disorder D-peak which is attributed primarily to amorphous carbon [29, 31], whereas the taller G-peak at ~ 1600 cm^−1^ is attributed to graphitic carbon, which in this case would be the CNTs themselves. The presence of a strong G’ peak at ~ 2650 cm^−1^ combined with an I_G_/I_D_ of under 1 is also indicative of multi-walled CNTs in the product, which is in line with previous work [23, 24]. Likewise, we do not see radial breathing modes at 785 nm, as seen in Fig. 5b, where most CNTs would resonate at. The spectra shown here are averaged over multiple acquisitions and are representative of the entire CNT ensemble. ESI figure S2 shows a representative Raman spectrum collected for the control experiment with no RBMs and a subdued G’ peak to further show there are not only no SWCNTs there, but also very few CNTs in the absence of face masks as feedstock.

**Fig. 6:**
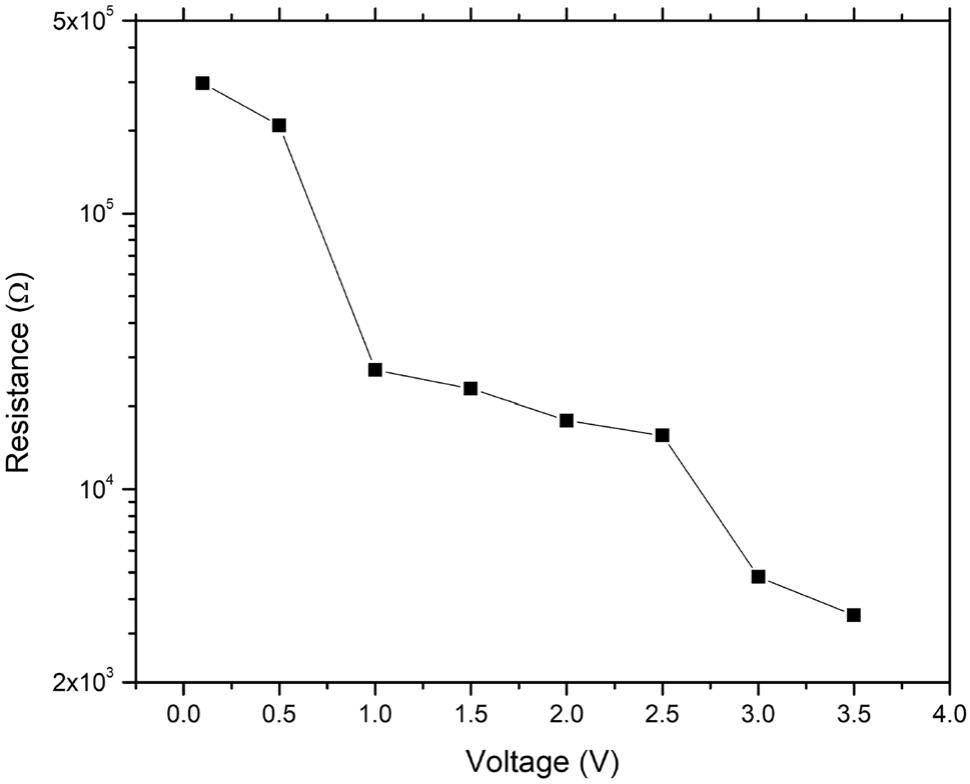
Electrical resistance of CNTs produced from face mask materials as a function of applied voltage in air. The resistance drops continuously with voltage until 3.5 V followed by which the conduction pathway was disrupted by CNT oxidation

Electrical testing was done in air, meaning there was already a limit present on how much voltage could be run through the make-shift CNT-based conductor before amorphous carbon and/or CNTs oxidized, and the conduction pathway disrupted [32]. Figure 6 shows a multi-stage decrease in electrical resistance with applied voltage, which acts analogous to the voltage annealing of CNT fibres previously reported [33] with the removal of surface contaminants and amorphous carbon first before irreversible damage to the CNTs just past 3.5 V. A sealed conductor composed of aligned and purified CNTs [33, 34] should be able to withstand more and have a lower electrical resistance as well, but the goal here is to show these are CNTs that were manufactured from face mask materials and are electrically conductive. A second such test was done on a new sample with an increasing voltage sweep to 3 V and then decreasing back to 1 V, as seen in ESI figure S3, and we see a permanently lowered resistance owing to the removal of the impurities and an increased electrical conductance of the pathway now dominated by CNTs. The absolute numbers here are a function of many factors, including contact resistances and packing efficiency of the CNTs, as well as the 3-dimensional electrical conduction pathways. As such, it is not recommended to compare these numbers to any other measurements obtained separately.

### CNT Ethernet cable

Given the CNTs were electrically conductive, the next step involved the production of a device for applications that go beyond the lab. In this case, we produced an Ethernet cable as discussed in the material and methods section, and iPerf3 was used to quantify its performance. Table 1 shows the maximum uplink and downlink speeds in Mbps of the CNT cable compared to a control with only copper wiring manufactured the same way, and another control in the form of a commercially available Ethernet cable certified to hit CAT6 standards. The results show the CNT-based Ethernet cable can reach ~ 100 Mbps throughput to meet CAT5 transmission standards, and these speeds are higher than the UK adoption and Broadband classification as determined by the UK Government regulator [23]. The control experiments show that the copper wire gauge used, as well as the RJ45 connectors, are holding back the potential of the CNT wires themselves as determined by the fact Cu wires, once connected to identical RJ45 connectors, also maxed out at the same values. Industrial grade RJ45 connectors certified for CAT6 standards coupled with higher quality, thicker gauge copper wiring as well as better assembly and crimping will help negate the bottleneck. A video showing an example iPerf test is available online [35].

**Table 1:** Ethernet transmission tests using three cables including a commercial CAT6 device and two lab made cables using CNTs or Cu wires as the active transmission component

	Maximum uplink speed (Mbps)	Maximum downlink speed (Mbps)
Cat6	998	995
Cu cable	92.7	94.7
CNT cable	94.9	97

### Global energy considerations

In considering the use of plastics as a feedstock we often encounter questions surrounding the energy costs of using plastics compared to not using them. The only difference in energy conversion from toluene only to toluene+face masks came in the form of the dissolution step itself, which we acknowledge effectively takes 30 min only at 85 °C, although we left it ongoing for 10 h to be prudent. The 10 h scenario would result in an energy consumption of 1.2 kWh based on repeated experiments done, and that corresponds to an energy cost of 12p based on a fixed industrial contract rate of 10 p/kWh available in the UK, or ~ 25p based on current consumer variable rates. Our findings for the control experiments with toluene only also reveals a 75% increase in product yield, most of which are CNTs, as opposed to predominantly amorphous carbon with toluene only. Given that CNTs currently sell for $100–150/g, the cost of the mixed solution being converted to CNTs is significantly better value than simply using toluene alone. Secondly, while there are several different routes to dispose of face masks, we contacted a few different recycling and waste management companies to get a better understanding of their energy costs and requirements. Overall, it is extremely hard to calculate the exact cost of disposing a single face mask, with some estimates in the range of 3–5p for simply the fuel used for burning and further clean-up, and others going as high as 35p when accounting also for human labour costs and transportation to a landfill or even to other countries. That said, we also need to recognize the additional environmental costs of face mask disposal on top of the economic cost. Overall, we are convinced that our process is extremely green in not only resource consumption but also product value generation as opposed to waste creation. One thing is certain, there is plenty of plastics available with more becoming continually available and in a macroeconomic context we don’t know how to handle them (or else we wouldn’t have a growing plastics crisis) but, we have a process that upcycles plastics in our open-loop chemical recycling process to a material that is orders of magnitude more valuable than the plastics themselves, therefore in the first instance until we have as a society developed all the solutions to stem the tide of our plastic crisis we might as well start with making the plastics into carbon nanotubes.

## Conclusions

This work began as an exploration of upcycling different plastics beyond polystyrene for the synthesis of carbon nanotubes. Collaboration with another research group led to the procurement of face mask materials that use a polyester base that is then treated to have improved efficacy as a face mask for the capture of bacterial and viral content. The material used is an analogue to commercially available face masks that have been produced, used, and disposed of in vast quantities over the last 3 years, with the goal being to have a scalable process to convert the waste that costs money to dispose of (collection, transportation, landfill/burning, clean up, etc.) to a value-added product instead. A liquid dispersion of the face mask material in toluene was found to be easy to create, with the overall energy consumption of the dissolution phase minimal compared to the product value achieved relative to simply using toluene without the face masks. This dispersion was then injected into a chemical vapor deposition reactor under optimized growth conditions for the synthesis of carbon nanotubes. The solid product formed showed to have a mix of single- and multi-walled tubes. These CNTs were also shown to be electrically conductive, and an Ethernet cable produced using them was shown to adhere to CAT5 transmission speeds while easily exceeding the benchmarks set for Broadband internet in most countries. There is scope for improvement in many places, but this is a crucial piece of work contributing to not only a circular economy but is also scalable and is viable for industrial processing with green chemistry at its core.

## Supplementary Information

Below is the link to the electronic supplementary material.

Supplementary file1 (DOCX 409 KB)
