# Supplementary material for: Upcycling of face masks to application-rich multi- and single-walled carbon nanotubes
Source: Carbon Lett (Korean Carbon Soc). 2022 Sep 30;32(7):1681–8. doi: 10.1007/s42823-022-00398-8 (PMC9523627; doi:10.1007/s42823-022-00398-8)
Supplement: Supplementary file 1 — Supplementary file1 (DOCX 409 KB) [file 42823_2022_398_MOESM1_ESM.docx]

Upcycling of Face Masks to Application-rich Single- and Multi-Walled Carbon Nanotubes

Varun Shenoy Gangoli,*^a,b^ Thomas Mahy, ^a^ Tim Yick, ^a^ Yubiao Niu,^c^ Richard E. Palmer^c^ and Alvin Orbaek White *^a,b^

1. Energy Safety Research Institute, Swansea University Bay Campus, Swansea, SA1 8EN, UK; [v.s.gangoli@swansea.ac.uk](mailto:v.s.gangoli@swansea.ac.uk) (V.S.G.); [920125@Swansea.ac.uk](mailto:920125@Swansea.ac.uk) (T.M.); [748963@Swansea.ac.u](mailto:748963@Swansea.ac.u)k (T.Y.); [alvin.orbaekwhite@swansea.ac.uk](mailto:alvin.orbaekwhite@swansea.ac.uk) (A.O.W.)
2. Faculty of Science and Engineering, Swansea University Bay Campus, Swansea, SA1 8EN, UK; [v.s.gangoli@swansea.ac.uk](mailto:v.s.gangoli@swansea.ac.uk) (V.S.G.); [alvin.orbaekwhite@swansea.ac.uk](mailto:alvin.orbaekwhite@swansea.ac.uk) (A.O.W.)
3. Nanomaterials Lab, Mechanical Engineering, Faculty of Science and Engineering, Swansea University, Bay Campus, Swansea, SA1 8EN, UK; [R.E.Palmer@Swansea.ac.uk](mailto:R.E.Palmer@Swansea.ac.uk) (R.E.P.); [Yubiao.Niu@Swansea.ac.uk](mailto:Yubiao.Niu@Swansea.ac.uk) (Y.U.)

* Correspondence: [v.s.gangoli@swansea.ac.uk](mailto:v.s.gangoli@swansea.ac.uk) (V.S.G.); [alvin.orbaekwhite@swansea.ac.uk](mailto:alvin.orbaekwhite@swansea.ac.uk); Tel.: +44-01792-604947 (A.O.W.)


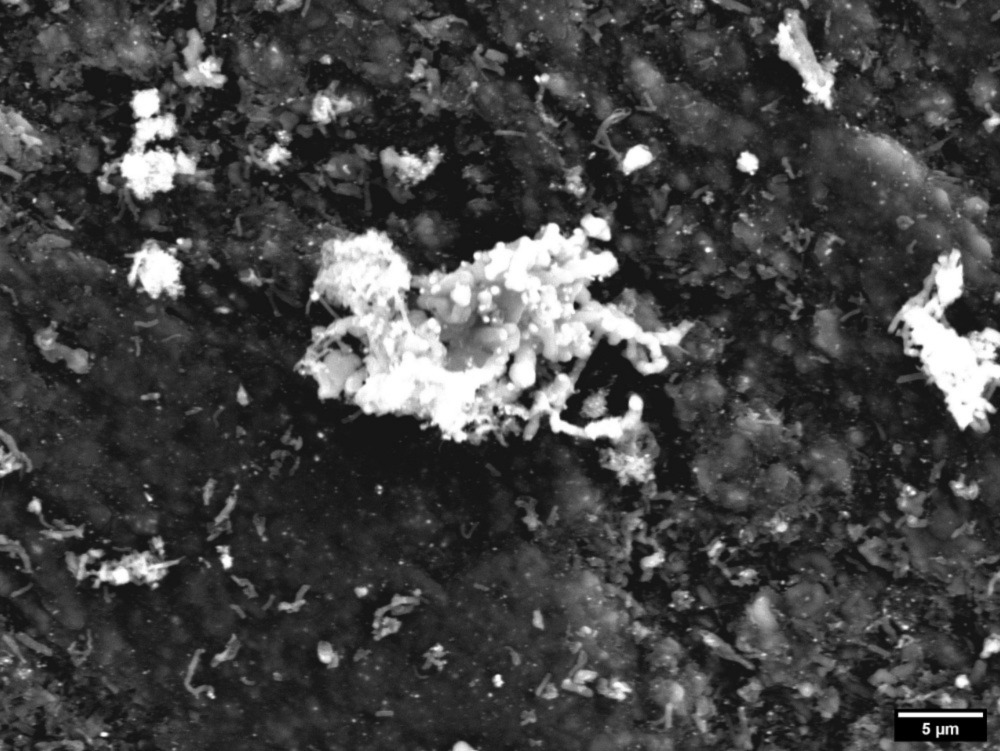


**Figure S1**. SEM image of product synthesized using just toluene and ferrocene injected into the CVD reactor without any face mask materials added. The product yield is not only much lower than with the face masks, but it is also predominantly amorphous carbon.


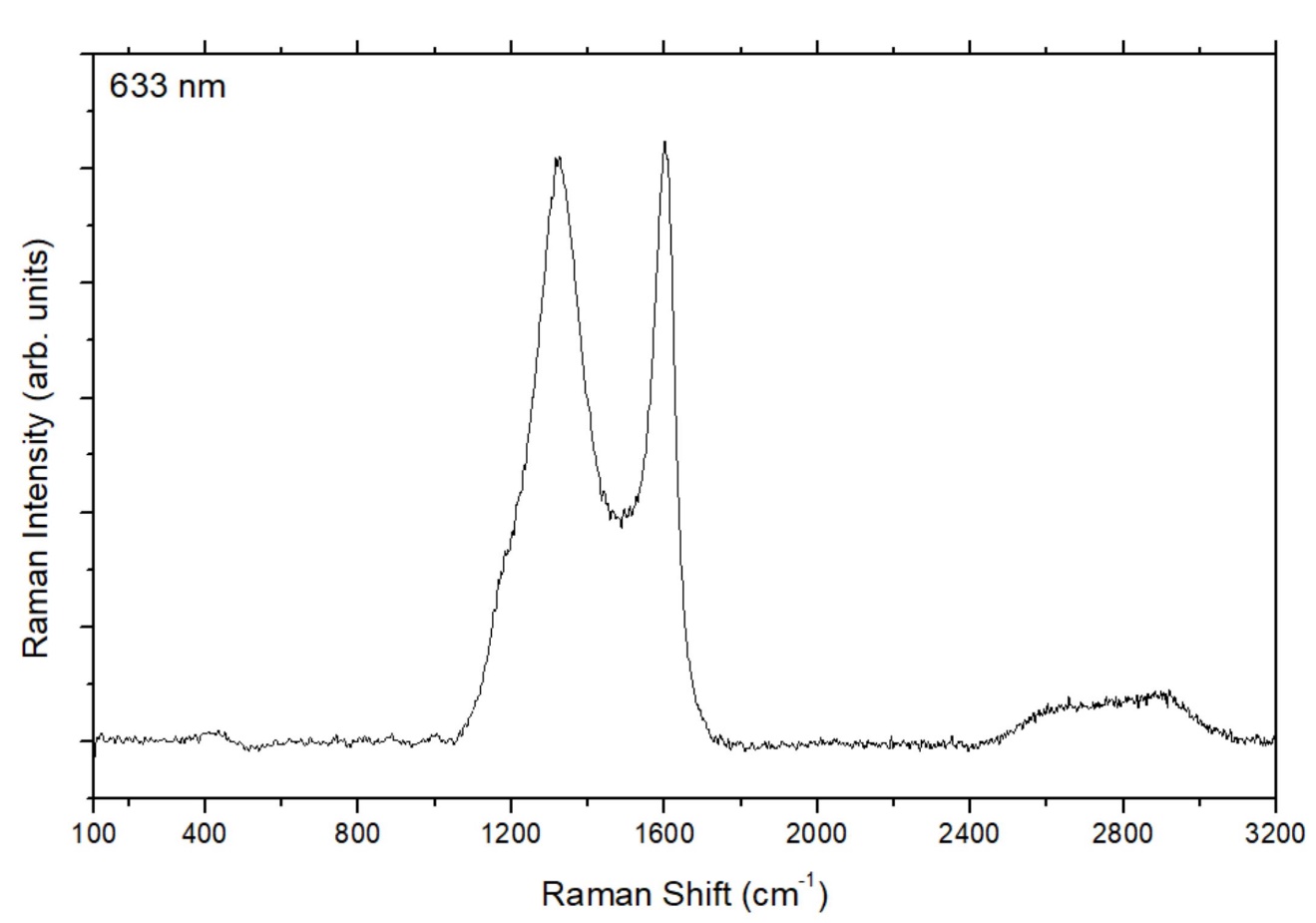


**Figure S2**. Raman spectroscopy at 633 nm of product synthesized using just toluene and ferrocene injected into the CVD reactor without any face mask materials added. The absence of any radial breathing modes as well as a subdued G’ peak here indicates the product is mostly comprised of amorphous carbon, with SEM imaging also indicating the presence of graphene shells and short carbon nano fibrils contributing to the G-peak here.


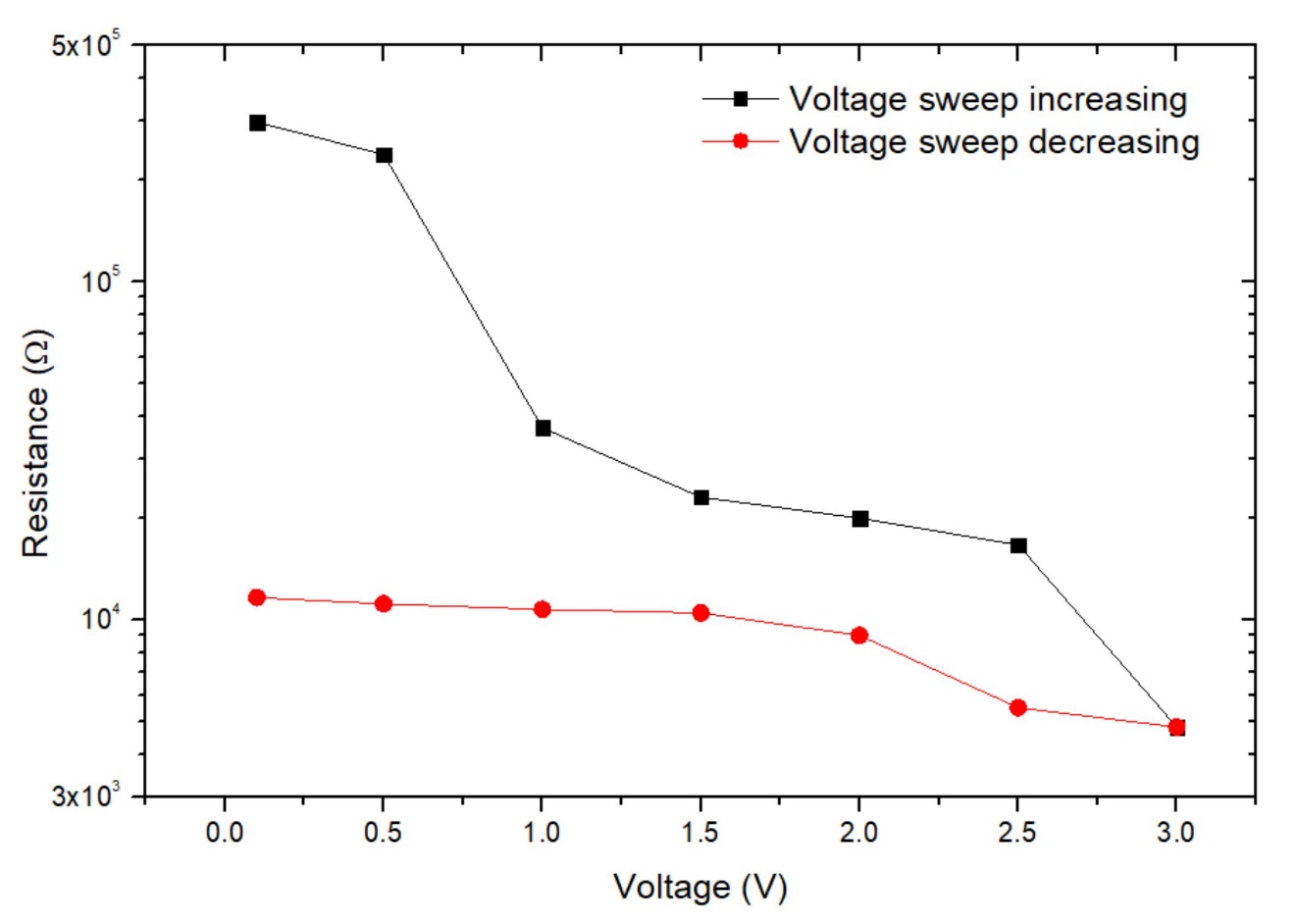


**Figure S3**. Electrical resistance of CNTs produced from face mask materials as a function of applied voltage in air. The resistance drops continuously with voltage until 3 V when doing an increasing voltage sweep followed by a voltage sweep in the decreasing direction that shows a permanently lowered resistance owing to the now cleaner and more electrically conductive pathway present.
